# The hippocampus underlies the association between self-esteem and physical health

**DOI:** 10.1038/s41598-018-34793-x

**Published:** 2018-11-20

**Authors:** Huanhua Lu, Xueting Li, Yinan Wang, Yiying Song, Jia Liu

**Affiliations:** 1 School of Marxism, China University of Geosciences (Beijing), Beijing, China; 2 Department of Psychology, Renmin University of China, Beijing, China; 3 Beijing Key Laboratory of Applied Experimental Psychology, National Demonstration Center for Experimental Psychology Education (Beijing Normal University), Faculty of Psychology, Beijing Normal University, Beijing, China

## Abstract

Self-esteem refers to the extent to which we feel positive or negative about ourselves, and reflects an individual’s subjective evaluation of personal worth and attitudes about the self. As one kind of positive psychosocial resources, high self-esteem has been found to buffer the effects of stress on physical health. However, little is known about the possible neural basis underlying the association between physical health and self-esteem. In the present study, we investigated whether the hippocampus served as a neuroanatomical basis for the association between self-esteem and physical health in a large population of healthy young adults. We examined self-esteem and self-reported physical health with the Rosenberg Self Esteem Scale (RSES) and the Chinese Constitution Questionnaire (CCQ) respectively, and gray matter volume of the hippocampus was measured using magnetic resonance imaging. As expected, we found that individuals with higher levels of self-esteem had better self-reported physical health. Importantly, the mediation analysis showed that the gray matter volume of the hippocampus mediated the link between self-esteem and physical health, suggesting its critical role in the neural circuitry through which self-esteem is related to physical health.

## Introduction

In our daily life, we all have a mental picture of who we are, how we look, what we’re good at, and what our weaknesses might be, which contributes to our self-esteem. Self-esteem is an overall reflection of an individual’s self-worth, and represents the capacity to feel worthy of happiness and be able to successfully address life challenges^1,2^. As a kind of important psychological resources, self-esteem is thought to be a protective factor for our physical health. Specifically, low self-esteem is accompanied by negative emotions such as anxiety and depression^3,4^, leads to unhealthy behaviors including smoking and alcohol abuse^5,6^, and then this combination increases the risk of heart diseases and other illness^7^. On the contrary, a high level of self-esteem is associated with positive feelings such as joy, pleasure, optimism, relaxation, and gratitude, which offer protection from stress and contribute to physical health^3,8,9^. In fact, many studies have demonstrated that higher self-esteem is related to better physical health and longer survival^2,10–13^. Several theories have been proposed to account for the cognitive mechanisms that link self-esteem to physical health^13–16^. However, little is known about the underlying neural basis through which self-esteem is related to physical health.

Abundant evidence suggests that the hippocampus is the core area of self-esteem^17–23^. For example, Pruessner *et al*.^17^ found that self-esteem was related to hippocampal volume in both young and old adults. A voxel-based morphometry (VBM) study^18^ showed that the gray matter (GM) volume of hippocampus was positively correlated with self-esteem. A recent study using a large sample also found that higher self-esteem was linked with greater GM volume of hippocampus, which was the region with the highest correlation to self-esteem within the whole brain^19^. On the other hand, the damage to the hippocampus has been demonstrated to be associated with not only a variety of neurological and psychiatric disorders, but also some physical diseases^24,25^. For example, studies of Type 2 diabetes have revealed reduced hippocampal volume^26^, hypertension was associated with hippocampal atrophy^27^. What’s more, the hippocampus shrinks in the pre-disease conditions, such as chronic jet-lag^28^ and elevated circulating inflammatory cytokines^29^. Meanwhile, greater hippocampal volume is associated with aerobic fitness which may improve our physical health^30^. In addition, in animal studies, hippocampal stimulation decreases heart rate, blood pressure and respiratory rate in awake rats^31^. Moreover, some recent studies have suggested the neural substrates that may underlie the interactions between psychological variables and physical health^32–34^. For example, Song *et al*. (2015) has shown that the amygdala underlies the association between emotion regulation and physical health. Similarly, recent work by Brody and colleagues has shown that enhancing supportive parenting can not only prevent the decline in volume of the hippocampus and amygdala but also reduce the incidence of prediabetes^32,33^. Therefore, we hypothesized that the hippocampus might serve as the neural substrate through which self-esteem is associated with physical health.

To test this hypothesis, we first examined the association between self-esteem and self-reported physical health in a large population of healthy young adults (N = 239) with two behavioral scales: the Rosenberg Self Esteem Scale (RSES)^1^ and the Chinese Constitution Questionnaire (CCQ)^35^ that is a national standard inventory on health in China. Then, we examined whether GM volume of the hippocampus mediated the relation between self-esteem and physical health, given that self-esteem is generally taken as a determinant of physical health not vice versa^13–16^.

## Material and Methods

### Participants

Two hundred and thirty nine college students (128 females; mean age = 22.8) from Beijing Normal University participated in this study. None of the participants reported history of neurological or psychiatric disorders. This study is part of an ongoing project (Gene Environment Brain and Behavior)^20,34,36^. Both the behavioral and fMRI protocol were approved by the Institutional Review Board (IRB) of Beijing Normal University and all experiments were performed in accordance with the relevant guidelines and regulations. All participants gave informed written consent prior to the experiments and then completed the paper-pencil questionnaires and structural MRI scanning. Because the acquisition of MRI data was time-consuming for the large sample of participants, the questionnaires were measured more than a month after MRI data acquisition.

## Experimental procedure

### Assessment of Self-Esteem

The self-esteem was assessed with RSES^1^, which contains ten items to assess one’s global self-worth incorporating both positive and negative feelings about the self (e.g., “On the whole, I am satisfied with myself”). Participants were instructed to report the extent to which they agreed or disagreed with each statement using a 6-point Likert-type scale (1 = strongly disagree, 6 = strongly agree). The total score of self-esteem was calculated by summing the score of each item, with higher scores indicating more positive evaluations of one’s worth and value.

### Assessment of physical health

Physical health was assessed with the CCQ^35^, which was designed to measure the general physical health status of individuals in normal population. It contains 59 items on a variety of physical symptoms and health problems (e.g., “I catch cold more easily than others.”). Participants were instructed to answer each question based on their daily experiences on a 5-point Likert-scale (1 = Never, 5 = All the time). To get a pure measure of physical health, eight items that are related to psychological symptoms such as “I get anxious and worried easily” were excluded in future analyses. The total score of physical health was calculated by summing the score of remaining 51 items, with higher scores indicating better physical health. Previous studies have shown that the questionnaire has high reliability and validity^35^. A recent study^34^ showed that participants’ physical health assessed by the CCQ was positively correlated with their general evaluation about their physical health status (r = 0.34, *p* < 0.001), indicating that the CCQ had good validity.

### MRI data acquisition and analysis

Structural MRI scanning was performed on a Siemens 3 T Trio scanner (MAGENTOM Trio with a Tim system) with a 12-channel phased-array head coil at BNU Imaging Center for Brain Research, Beijing, China. MPRAGE, an inversion prepared gradient echo sequence (bandwidth = 190 Hz/pixel, flip angle = 7 degree, TR/TE/TI = 2.53 s/3.39 ms/1.1 s), was used to acquire 3D T1-weighted whole brain structural images. One hundred and twenty-eight contiguous sagittal slices were imaged with 1 × 1 mm in-plane resolution and 1.33 mm slice thickness for whole brain coverage.

Voxel-based morphometry (VBM) which provides a quantitative measure of tissue volume per unit volume of spatially normalized image was used to explore the neural correlates of the behaviorally-observed association^37^. Specially, VBM was performed using SPM8 (Statistical Parametric Mapping, Wellcome Department of Imaging Neuroscience, London, UK). Preprocessing steps and an optimized VBM protocol were performed on T1-weighted structural images. First, image quality was assessed by manual visual examination. Five participants whose images had excessive scanner artifacts or showed gross anatomical abnormalities were excluded. Second, the origin of brain was manually set to the anterior commissure for each participant. Third, images were segmented into four distinct tissue classes: GM, white matter, cerebrospinal fluid and everything else (e.g., skull and scalp) using a unified segmentation approach^38^. Forth, the GM images for each participant were then normalized to a study-specific template in MNI152 space using the Diffeomorphic Anatomical Registration through Exponential Lie algebra (DARTEL) registration method^39^. The DARTEL registration involves iteratively computing the study-specific template, based on the average tissue probability maps from all participants, and then warping all participants’ tissue maps into the generated template to increasingly improve the alignment. Fifth, GM voxel values were modulated by multiplying the Jacobian determinants derived from the normalization to preserve the volume of tissue from each structure after warping. The modulated GM images were then smoothed with an 8 mm full width at half maximum (FWHM) isotropic Gaussian kernel. Finally, to exclude noisy voxels, the modulated images were masked using an absolute masking with a threshold of 0.2. The masked modulated GM images were used for further statistical analyses.

We used the Harvard-Oxford subcortical probabilistic structural atlas^40^ with a probability threshold of 50% to define the left and right hippocampus as regions of interest (ROI). We calculated averaged GM volume across all voxels in each ROI. To determine the neural correlates of behavioral measures, we performed Pearson correlation analyses between average GM volume of the left and right hippocampus and behavioral measures of self-reported physical health and self-esteem. Further, we performed stepwise regression analyses which took the total GM volume (TGMV) and gender as confounding covariates to confirm the results. Finally, we conducted a mediation analysis to examine whether the hippocampus served as a neuroanatomical basis for the association between self-esteem and physical health. Here bootstrap simulation was used to test the significance of the mediation effect. Because the sampling distribution of mediation effect is skewed, bootstrap approach has been proved to be more powerful than traditional methods (e.g., Sobel test) and has been widely used in mediation analysis in recent years^41,42^.

## Results

First, we used the RSES 1 and the CCQ^35^ to measure participants’ self-esteem and physical health respectively. The kurtosis (CCQ: −0.56, RSES: −0.42) and skewness (CCQ: −0.12, RSES: −0.28) of the two scales indicated the normality of the data, and both scales possessed excellent internal reliabilities (Cronbach’s α_RSES_ = 0.897, Cronbach’s α_CCQ_ = 0.92). The scores of both CCQ (mean = 187.41; SD = 44.78) and RSES (mean = 21.67; SD = 6.38) showed a significant amount of individual differences.

**Figure 1:**
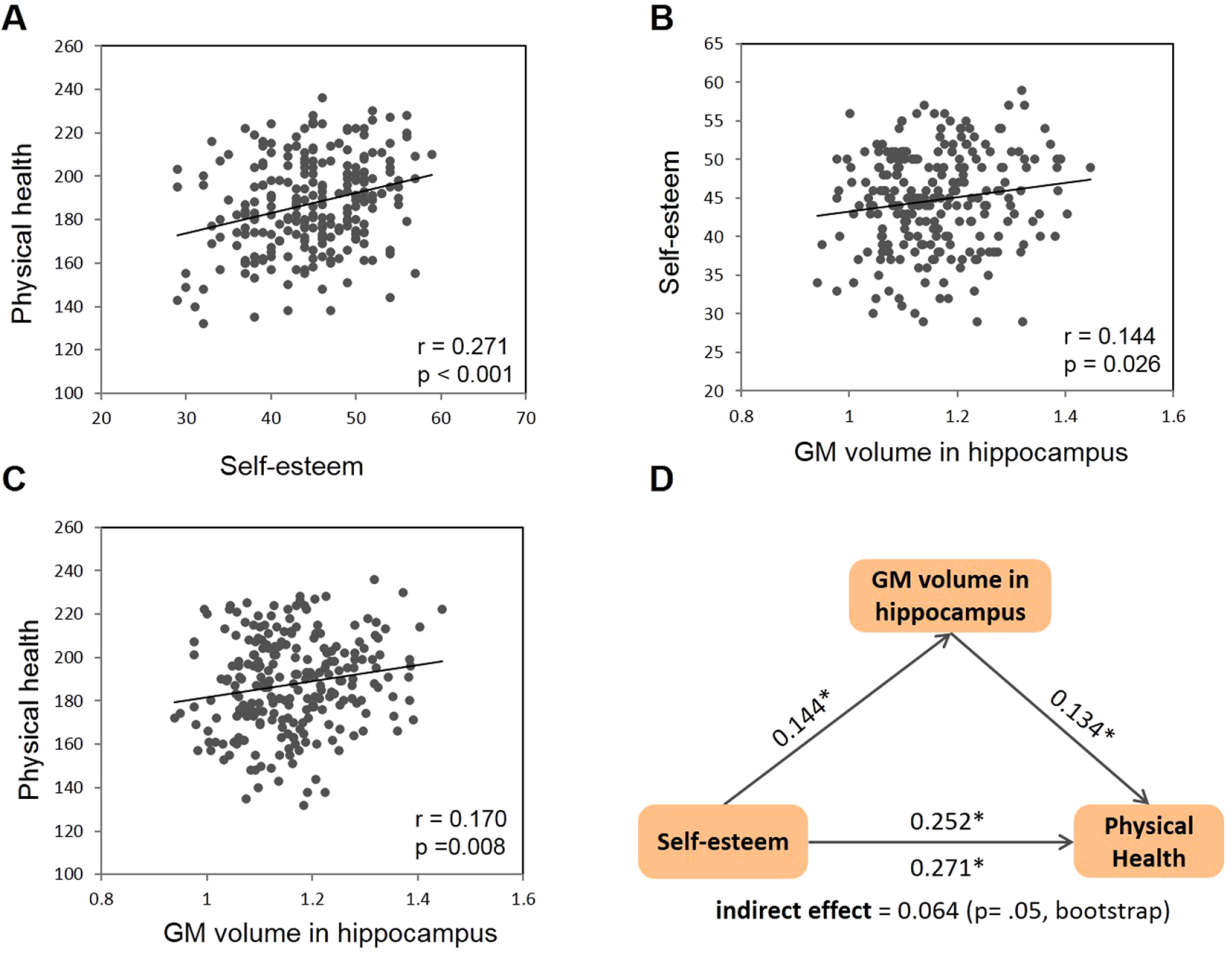
Relationship between gray matter (GM) volume in the hippocampus, self-esteem, and physical health. Scatter plots for correlation between (**a**) physical health and self-esteem, (**b**) self-esteem and average GM volume in the hippocampus, and (**c**) physical health and average GM volume in the hippocampus. (**d**) GM volume in the hippocampus as a mediator in the correlation between self-esteem and physical health in the mediation analysis. Path coefficients are shown next to arrows indicating each link in the analysis. For the association between self-esteem and physical health, the value below the arrow indicates the zero-order correlation, and the value above the arrow represents the correlation after controlling the mediator of GM volume in the hippocampus. All values represent standardized betas. *p < 0.01; **p < 0.001.

To investigate the relationship between self-esteem and physical health, we correlated self-esteem scores with self-reported physical health measured by the CCQ. The result showed that self-esteem was positively correlated with physical health (r = 0.271, *p* < 0.001; Fig. 1A), indicating that individuals with higher self-esteem possessed better physical health. The finding was further confirmed by a stepwise linear regression analysis which showed that self-esteem was significantly correlated with physical health with gender regressed out (β = 0.272, *p* < 0.001). Next, we investigated the neural basis through which self-esteem was associated with physical health.

Considering that self-esteem has been demonstrated to be related with the GM volume of hippocampus^17–20,22^ and the hippocampus is associated with some physical diseases such as hypertension and diabetes^26,27^, we hypothesized that the hippocampus might be the neural basis through which self-esteem is related to physical health. To examine this hypothesis, we first confirmed that the GM volume in both the left (r = 0.142, *p* = 0.028) and right hippocampus (r = 0.141, *p* = 0.029) was positively correlated with self-esteem, indicating that individuals with larger GM volume in the hippocampus possessed higher self-esteem. This association has been reported in our previous study using the same dataset^19,20^ as well as in several other studies^17,18,22^. Besides, the results also showed positive correlation between physical health and the GM volume in the left (r = 0.183, *p* = 0.005) and right hippocampus (r = 0.150, *p* = 0.021), suggesting that individuals with larger GM volume in the hippocampus had better physical health. The finding was further confirmed by stepwise linear regression analyses which showed that the GM volume in the left and right hippocampus was significantly related with individual’s self-esteem (β_left_ = 0.142, *p* = 0.028; β_right_ = 0.141, *p* = 0.029) and physical health (β_left_ = 0.183, *p* = 0.005; β_right_ = 0.150, *p* = 0.021) with TGMV and gender regressed out. Because similar results were observed in both hemispheres, the GM volume across the left and right hippocampus was collapsed (GM volume in the whole hippocampus and self-esteem: r = 0.144, *p* = 0.026, Fig. 1B; GM volume in the whole hippocampus and physical health: r = 0.170, *p* = 0.008, Fig. 1C).

Because individuals who had larger GM volume in the hippocampus possessed not only higher self-esteem but also better physical health, it is likely that the hippocampus serves as a possible neural substrate for the link between physical health and self-esteem. To test this hypothesis, a mediation analysis based on individual differences in self-esteem, physical health, and GM volume of the hippocampus was performed. The result showed that the correlation between self-esteem and physical health decreased (r = 0.252, *p* < 0.001) after their correlation (r = 0.271, *p* < 0.001) being adjusted by the mediator of the hippocampus (Fig. 1D). Bootstrap simulation (n = 5000) further confirmed that the indirect effect of self-esteem on physical health through the GM volume of the hippocampus was significant (*p* = 0.05) with 95% confidence interval (CI) of [0.007, 0.182]. The result suggests that self-esteem is related to physical health in part through the GM volume of hippocampus.

## Discussion

In the current study, we investigated the possible neural basis through which self-esteem was related to physical health. Our study not only reproduced the results that higher self-esteem was associated with better physical health, but also showed that the hippocampus mediated the link between self-esteem and physical health, suggesting its critical role in the neural circuit through which self-esteem is related to physical health.

Our study replicated previous findings that higher self-esteem is associated with better physical health^2,10–13,43^. Why is higher self-esteem link with better physical health? Several models used in health promotion or prevention offer insight into cognitive mechanisms that link self-esteem to health^13–16^. For example, the transactional model of stress and coping suggests that positive self-esteem can buffer stress by mitigating the perceived threat and by enhancing the selection and implementation of efficacious coping strategies, which in turn modulate physiological responses to stressors contributing to illness and physical health^14^. Generally, self-esteem can be seen as an internal moderator of stressors, and increasing the levels of self-esteem will reduce the incidence of illness^15^. Alternatively, the health behavior model has confirmed the role of self-esteem as a health behavioral determinant, that is, low self-esteem can trigger poor coping behavior or risk behavior that subsequently increases the likelihood of certain diseases, including mental disorders and physical diseases^16,44^. In fact, previous studies had verified that low levels of self-esteem was related to the incidence of health risk behaviors, such as smoking, alcohol consumption and drug use^5,6,45^, which may do harm to our health. Based on these models and evidences, self-esteem may be related to physical health by buffering the stress or increasing the health behaviors.

Our study showed that the GM volume of hippocampus was related to the individual difference in physical health in the healthy sample. The results extend the previous studies that have demonstrated the association between hippocampus volume and physical diseases such as hypertension and diabetes^26,27^. A large body of research has shown that the interaction of the hippocampus and the hypothalamic-pituitary-adrenal (HPA) axis might explain the onset of various illnesses^25,46–48^. The HPA axis has traditionally been regarded as the body’s stress tolerance and energy regulation system. Its hyperactivation is associated with excessive release of stress hormones (e.g. glucocorticoid, cortisol) and subsequently results in various health problems^25,47^. Meanwhile, the hippocampus activity has been suggested to exert a tonic inhibitory influence on the activation of HPA axis and subsequently decrease glucocorticoid secretion, and promote more efficient regulation of the HPA axis to ultimately promote physical health^48^.

Importantly, our study showed that self-esteem was associated with physical health through the hippocampus. This suggests that the hippocampus is a key node in the neural circuit underlying the link between self-esteem and physical health. In the face of stressful situations, the HPA axis, the autonomic nervous system, and the cardiovascular, metabolic, and immune systems interact with each other to achieve allostasis (adaptation) in the short term and lead to allostatic load (“the wear and tear on the body”) in the long term^49^. Allostatic load can result in altered brain architecture, especially in the hippocampus^49^. For example, there is evidence that prolonged stress which triggers the over release of glucocorticoids caused the atrophy of the hippocampus^50–57^. On the other hand, high self-esteem can help individuals to take effective coping strategies that buffers physiological responses to stressors^17,20,22,23^, and low self-esteem was related to higher cortisol response to stress task^58^ and lack of habituation to repeated stress exposure^59^. Consequently, high self-esteem can prevent the hippocampus from the harmful effects of allostatic load such as excessive glucocorticoids. Better preserved hippocampus in turn inhibits the prolonged excessive activity of the HPA, which can reduce allostatic load and guard against the disorders of neuroendocrine and immune systems, and ultimately promotes physical health^46,60^.

In sum, our study revealed the possible neural substrate underlying the association between physical health and self-esteem. Practically, given the protective role of self-esteem on physical health, it is recommended that its potential should be reconsidered in future health promotion programs. That is, a health promotion program should include a focus on fostering self-esteem, which can prevent an array of physical diseases. There are several issues unresolved in our study. First, the cross-sectional findings in the current study do not permit inference about the causal relationship among self-esteem, physical health, and the hippocampus, and future longitudinal studies are needed to provide better understanding for this topic. Secondly, we did not measure health behaviors in the current study and our results were inappropriate for providing neural constraints for the health behavior models. Therefore, future researches are needed to illuminate the neural mechanism through which physical health might be influenced by the interaction of self-esteem and health behaviors.
